# Hypercalcemic Crisis Complicated With Acute Respiratory Distress Syndrome Due to Hot Spring Drowning: A Case Report

**DOI:** 10.7759/cureus.58431

**Published:** 2024-04-16

**Authors:** Miyuki Hattori, Kazuya Kikutani, Shinichiro Ohshimo, Nobuaki Shime

**Affiliations:** 1 Department of Emergency and Critical Care Medicine, Hiroshima University Graduate School of Biomedical and Health Sciences, Hiroshima, JPN

**Keywords:** hemodialysis, acute respiratory distress syndrome, hot spring, drowning, hypercalcemia

## Abstract

Hypercalcemia is generally treated conservatively, including massive fluid administration. However, in cases of acute respiratory distress syndrome (ARDS) associated with drowning, excessive fluid administration may worsen respiratory status.

An 81-year-old female was found drowned in a hot spring at an accommodation facility and urgently transported to our hospital. On arrival, the patient exhibited severe respiratory failure, impaired consciousness, and bilateral lung infiltrates on computed tomography (CT), suggesting ARDS. Blood biochemical tests showed calcium (Ca) of 17.4 mg/dL, with altered consciousness attributed to hypercalcemia. Because of concerns about further deterioration of respiratory status, hemodialysis was performed to avoid massive fluid administration. Post-hemodialysis, blood calcium levels quickly decreased, leading to improved consciousness and respiration; the patient was extubated 48 hours post-admission. Subsequent examinations identified hot spring water aspiration as the cause of hypercalcemia.

For hypercalcemia from hot spring drowning with acute respiratory distress syndrome, consider early hemodialysis initiation without excessive fluid administration.

## Introduction

A hypercalcemic crisis is a life-threatening condition caused by hypercalcemia (serum calcium (Ca) ≥ 14 mg/dL), which induces systemic changes such as central nervous system damage and acute kidney injury [1].

The causes of hypercalcemia include primary hyperparathyroidism, characterized by excessive parathyroid hormone (PTH) secretion, vitamin D excess, PTH-related peptide (PTHrP) production, or local bone resorption by malignant tumors [2]. Additionally, medications, prolonged bed rest, and exogenous intakes such as milk-alkali syndrome and calcium sulfate beads [3] can also be contributing factors.

Drowning in a hot spring can cause a hypercalcemic crisis due to aspiration or accidental ingestion of hot spring water [4-6]. Hypercalcemia treatment is generally based on conservative therapy, including massive fluid administration, calcitonin, and bisphosphonates [7]. However, in cases of acute respiratory distress syndrome (ARDS) associated with drowning, massive fluid administration may exacerbate lung damage. Here, we report a case of drowning in hot spring water that manifested as a hypercalcemic crisis and ARDS, successfully managed through prompt initiation of hemodialysis.

## Case presentation

**Figure 1:**
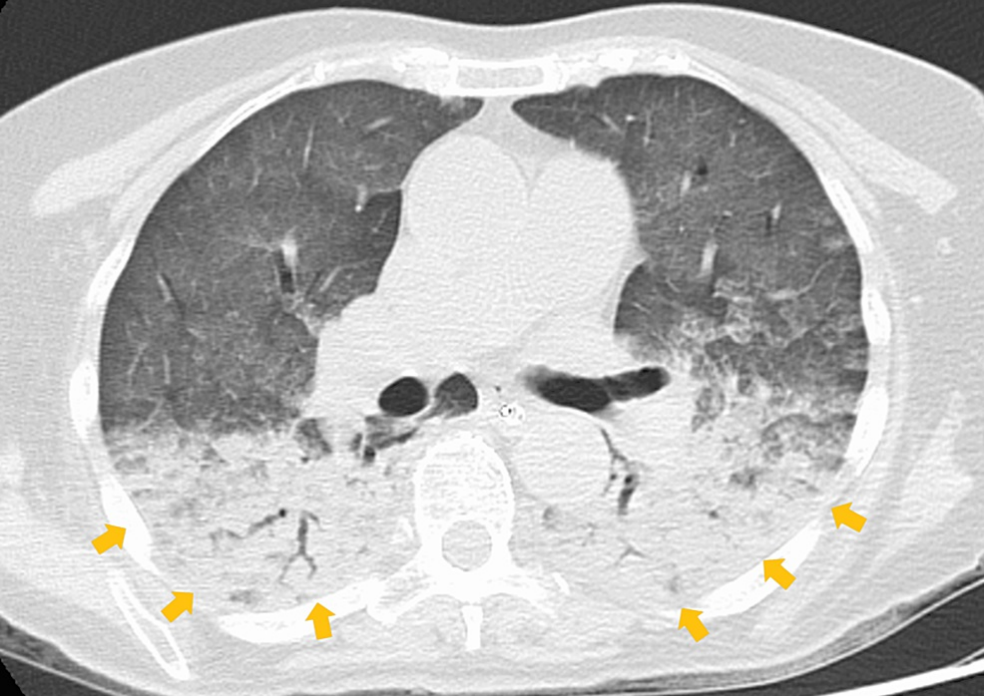
Computed tomography showing bilateral lung infiltrates Chest HRCT shows bilateral infiltrating shadows with air bronchograms in the dorsal lungs (arrows). Both lungs also showed diffuse ground-glass attenuation due to gravity. No cardiac enlargement was observed. These findings are consistent with those of ARDS due to drowning. HRCT: high-resolution computed tomography, ARDS: acute respiratory distress syndrome

An 81-year-old female was urgently transported to our hospital after drowning in a hot spring. The patient was discovered drowning in a bathtub about an hour after the last confirmation of their well-being, but the precise time of drowning remained unclear. Upon arrival at the emergency room, the patient's consciousness level was Glasgow Coma Scale 3, with arterial oxygen saturation at 90% (under 15 L/minute oxygen administration), respiratory rate of 18 breaths/minute, blood pressure of 237/56 mmHg, heart rate of 114 beats/minute, and body temperature of 38.4°C. Emergency endotracheal intubation was performed due to impaired consciousness and severe hypoxemia. Computed tomography (CT) scan revealed bilateral lung infiltrates (Figure 1). The brain CT scan showed no evidence of diseases causing impaired consciousness. Arterial blood gases were pH 7.29, partial pressure of carbon dioxide (pCO2) 47.6 mmHg, partial pressure of oxygen (pO2) 107 mmHg, bicarbonate (HCO3-) 22.3 mmol/L, lactate 3.4 mmol/L, and fraction of inspired oxygen (FIO2) 1.0 (P/F ratio: 107). Blood biochemistry data showed serum total calcium of 17.4 mg/dL, ionized calcium of 2.41 mmol/L, albumin of 4.7 g/dL, magnesium of 3.8 mg/dL, sodium of 139 mmol/L, potassium of 3.1 mmol/L, phosphorus of 4.1 mg/dL, urea nitrogen of 16.3 mg/dL, and creatinine of 0.68 mg/dL. Electrocardiography and echocardiography revealed no signs suggestive of myocardial infarction or heart failure. Based on the patient's history and examination results, she was diagnosed with altered consciousness due to hypercalcemia and hypoxemia caused by ARDS resulting from drowning.

Massive fluid administration, a common treatment for hypercalcemia, was not performed in this case, considering the risk of worsening respiratory status. Instead, intermittent hemodialysis (blood flow: 150 mL/minute, flow rate of dialysate: 500 mL/minute, membrane area: 1.5 m², time: 4 hours) was performed to relieve severe hypercalcemia. After one session of dialysis, the ionized serum calcium level decreased to 1.59 mmol/L. Lung-protective mechanical ventilation was initiated with the patient in the prone position. Twenty-four hours after admission, the respiratory status improved to a P/F (PaO2/FIO2) ratio of 364, and bilateral lung infiltrates markedly improved on chest radiography. As the serum calcium level did not increase again and the level of consciousness and respiration gradually improved after discontinuing intravenous anesthesia, the patient was successfully extubated 48 hours after admission.

No relevant causes for hypercalcemia were detected in the patient's medical history or current medications. Serum PTH was intact at 12 ng/mL, and PTHrP was within the normal range. Hypervitaminosis was not observed with 1,25(OH)VitD 14 pg/mL and 25(OH)VitD 15.8 ng/mL. Whole-body CT scan revealed no obvious findings suggestive of malignancy or sarcoidosis. The source of the hot spring water where the patient drowned was high in calcium ions at 1,233 mg/kg (31 mmol/L); thus, we concluded that the patient's hypercalcemia was caused by accidental aspiration of the hot spring water.

## Discussion

In Japan, with its strong bathing culture, approximately 19,000 bathing-related deaths occur annually. Drowning in bathtubs is considered a public health problem, especially among older adults [8]. In drowning, when accompanied by massive aspiration, electrolyte abnormalities may occur depending on the electrolyte content of the water. This is because natural hot spring water may contain many minerals, particularly calcium. To the best of our knowledge, only three reports in English have documented hypercalcemia due to drowning in hot springs, all originating from Japan (Table 1) [4-6].

**Table 1:** Previous reports of hypercalcemia due to hot spring drowning ARDS: acute respiratory distress syndrome, MV: mechanical ventilation

Study	Age	Sex	Treatment for hypercalcemia	Tracheal intubation	ARDS	Duration of MV (day)
Matsumoto et al. [4]	66	Male	Continuous hemodiafiltration	Yes	Insufficient information	No information
Machi et al. [5]	73	Male	Fluid loading	No	No	-
Ueno et al. [6]	55	Female	Fluid loading and calcitonin	Yes	Insufficient information	11
Our case	81	Female	Hemodialysis	Yes	Yes	2

It has been reported that hypercalcemia can occur with drowning in specific regions such as the Dead Sea and can be fatal due to electrolyte abnormalities caused by seawater aspiration [9]. The quality of hot springs in Japan varies widely, and even within the same region, the minerals they contain can differ significantly [10]. In cases of drowning in hot springs with electrolyte abnormalities, confirming the hot spring analysis report may be necessary to investigate the cause.

While both aspiration and ingestion are considered as potential routes for calcium absorption from the hot spring water, considering the faster absorption from the alveoli compared to the gastrointestinal tract, it was highly likely that, similar to previous reports [4,5], calcium was absorbed from the alveoli. Additionally, the fact that watery contents of the stomach and intestines were not prominent on the CT scan further suggested the possibility of absorption from the alveoli.

This is the first report of a hypercalcemic crisis caused by drowning in a hot spring, complicated with ARDS, which was definitively diagnosed, requiring ventilation. The typical regimen for the treatment of hypercalcemia is a 1-2 L bolus of normal saline solution, followed by 200-250 mL/hour [11]. However, a restrictive infusion strategy is recommended for patients with ARDS because of the risk of exacerbation of respiratory failure associated with massive infusion [12]. Although the amount of saline needed to treat the hypercalcemia was unclear, the patient probably required several liters of infusion. This high infusion volume may have had a negative effect on the improvement of ARDS in this patient. Unlike the common practice of massive fluid administration for hypercalcemia, as often reported in previous studies [5,6], in this case, due to the presence of consciousness disorders due to hypercalcemia and ARDS, renal replacement therapy (RRT) was chosen to promptly correct the serum calcium level and avoid worsening ARDS through fluid administration. In contrast to the report by Matsumoto et al. [4], in which continuous RRT was utilized to correct high calcium levels and achieved normalization of calcium levels within three days, our approach utilizing intermittent RRT may have facilitated rapid correction of calcium and potentially led to early extubation. Consequently, the patient quickly regained consciousness and was weaned off the ventilator early without worsening the lung condition.

## Conclusions

Drowning in hot springs can lead not only to respiratory failure but also to hypercalcemia depending on the quality of the spring water. The common treatment for hypercalcemia, massive fluid infusion, may lead to worsening of respiratory status in patients with respiratory failure. Our findings suggest that in cases of hot spring drowning presenting with ARDS complicated by severe hypercalcemia, early introduction of hemodialysis instead of massive fluid infusion may avoid delayed ARDS recovery and prolonged mechanical ventilation duration.
